# Elk population dynamics when carrying capacities vary within and among herds

**DOI:** 10.1038/s41598-020-72843-5

**Published:** 2020-09-29

**Authors:** Lisa J. Koetke, Adam Duarte, Floyd W. Weckerly

**Affiliations:** 1 Department of Natural Resources and Environmental Studies, University of Northern British Columbia, 3333 University Way, Prince George, BC V2N4Z9 Canada; 2 Oregon Cooperative Fish and Wildlife Research Unit, Department of Fisheries and Wildlife, Oregon State University, 104 Nash Hall, Corvallis, OR 97331 USA; 3 Department of Biology, Texas State University, 601 University Drive, San Marcos, TX 78666 USA

**Keywords:** Population dynamics, Theoretical ecology

## Abstract

Population and land management relies on understanding population regulation and growth, which may be impacted by variation in population growth parameters within and among populations. We explored the interactions between variation in carrying capacity (*K*), intrinsic population growth rate (*r*), and strength of density dependence (*β*) within and among elk (*Cervus elaphus*) herds in a small part of the geographic range of the species. We also estimated stochastic fluctuations in abundance around *K* for each herd. We fit linear Ricker growth models using Bayesian statistics to seven time series of elk population survey data. Our results indicate that *K* and *β* varied among herds, and that *r* and *β* varied temporally within herds. We also found that herds with smaller *K* had less stochastic fluctuation in abundances around *K*, but higher temporal variation in *β* within herds. Population regulation and the rate of return to the equilibrium abundance is often understood in terms of *β*, but ecological populations are dynamic systems, and temporal variation in population growth parameters may also influence regulation. Population models which accommodate variation both within and among herds in population growth parameters are necessary, even in mild climates, to fully understand population dynamics and manage populations.

## Introduction

Population regulation is a central concept in population ecology, and an understanding of how animal populations are regulated is vital to implement effective management and conservation decisions. Regulation of populations is driven by density-dependent factors, the strength of which should impact the time to return to an equilibrium abundance around which the population fluctuates^1^. This results in a carrying capacity (*K*) which can be defined as a long-term stationary probability distribution of population abundance^2–5^. This equilibrium can be driven by food limitation alone, or in combination with other sources of mortality, such as predation. The factors influencing population dynamics, such as the intrinsic population growth rate (*r*), strength of density dependence (*β*), and stochastic temporal variation in population abundance, may vary depending on *K*^6^ or independently of *K*^7^. Consequently, estimating *K*, *r*, temporal variation in *K* and *r*, and fluctuations in abundances around *K* is critical for understanding population dynamics and regulation^6,8^.

The Ricker model is commonly used to approximate population dynamics and estimate population growth parameters for species with slow life histories^9–11^. This model predicts a linear growth response such that *r* will decrease in a linear fashion as abundance increases. The Ricker model can be written as the following discrete equation^11,12^:

1 $$ N_{t + 1} = \alpha N_{t} e^{{ - \beta N_{t} }} , $$

where $N$ is the population abundance and *t* is time (year). In order to estimate the population parameters of interest, this basic equation can be linearized as:

2 $$ \ln \left( {\frac{{N_{t + 1} }}{{N_{t} }}} \right) = r_{t} = \ln \left( \alpha \right) - \beta N_{t } + \varepsilon , $$

where $\ln \left( \alpha \right) = r_{max}$, *r*_*max*_ is the biological maximum *r* for the species, *Ɛ* is the normally distributed residual variance, and *K* is the population abundance when *r* = 0. Although time series of population survey data from free ranging populations can indicate a non-linear relationship between abundance and population growth rate^10,13^, population survey data are often too noisy to distinguish between linear (Ricker) and non-linear (e.g., θ-logistic) population growth models^14^. Furthermore, the θ-logistic model has a number of disadvantages, including ridges and multiple peaks in the likelihood profile of *r*_*max*_ and θ, which can result in biologically implausible and imprecise estimates of these parameters^15–17^. Thus, the simplicity and parsimony of the Ricker model make it an insightful approximating model^14–17^.

The implications of using the Ricker model to understand temporal fluctuations in population abundance due to life history habitat, or population dynamics (i.e., process variance) have not been evaluated fully. Process variance is influenced by both demographic and environmental stochasticity^6,18^. Environmental variance stems from environmental changes over time. It includes density-dependent effects, which are biotic effects where the population growth rate depends on past or present abundance, and density-independent effects, which capture the impacts on the population growth rate of resource variation and abiotic conditions^8^. Demographic variance is random changes in demographic rates such as survival and fecundity, and its influence will decrease as population abundance increases.

Most current applications of the Ricker model assume that density-independent effects act on *r* and *N* simultaneously^9^. As such, density-independent effects are often assumed to have an additive relationship between *r* and *N*. Thus, the additive model captures the effects of density-independent environmental factors through temporal variation in *r*, but assumes no variation in *β*.

The multiplicative model, on the other hand, incorporates density-dependent effects while assuming little to no influence of density-independent factors on abundances. The multiplicative model approximates density-dependent effects by assuming a multiplicative relationship between *r* and $N$. Thus, the multiplicative model assumes that *K* varies temporally, and as such, the slope of the growth response, which estimates *β*, will vary.

In populations where density dependence can be detected, most have been explained by either the additive (i.e., temporal variation in *r*_*max*_) or multiplicative (i.e., temporal variation in *β*) model^9^. Nonetheless, populations do not necessarily follow either framework. Indeed, it is possible that population dynamics display no temporal variation in either parameter or temporal variation in both parameters^19,20^.

Variation in these parameters have been examined among^18^ or within populations^21^. Nonetheless, both among- and within-population variation can influence fluctuations in abundance around *K*. Therefore, an examination of the effect of variation in *K* both within and among populations on fluctuations in abundance around *K* is necessary. Furthermore, population growth parameters are often estimated over a substantial part of a species’ geographic range^18,22,23^, where environmental heterogeneity tends to be large, and so is variation in *K*. Two studies, however, indicate considerable variation in *K* within a small part of a species’ geographic range^7,24^, so variation in *β* and process variance at this spatial scale seems plausible. Thus, an examination of population dynamics within and among populations in *r* and *K* over a smaller part of the geographic range of a species is warranted.

We examined seven populations of elk (*Cervus elaphus*) inhabiting northern California and south-central Washington to explore the effect of variation in *r* and *K* within the framework of the additive and multiplicative models. Among populations, we hypothesized that *K* and *β* would vary among populations. Within populations, we hypothesized that temporal variation will not be detected in either *r* or *K* due to the stable composition of habitats and mild climate. We also estimated the influence of demographic and environmental stochasticity within each population. We hypothesized that in populations with smaller *K*, there will be less fluctuation in abundance around *K* due to strong density dependence. By exploring these hypotheses, our research assesses the influence of variation in *K* both among and within populations on their dynamics, regulation, and stability. These are important to fully understand, especially in the face of contemporary changes in the environment^5,8^. Thus, our findings regarding the impacts of *K* and *β* on population regulation and fluctuations in abundances around *K* have implications for population and land management.

## Results

**Figure 1:**
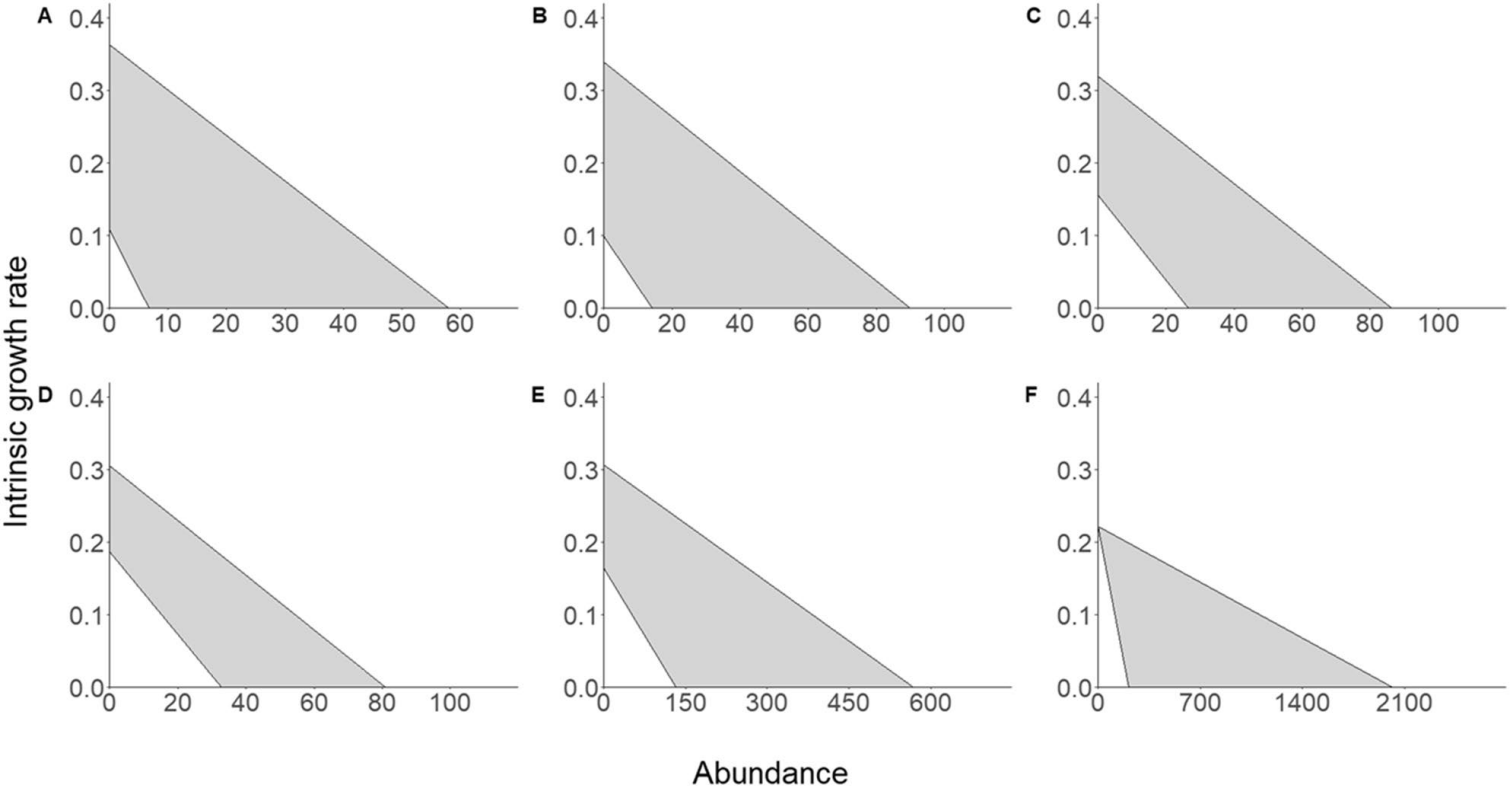
The fitted Ricker growth models for six elk herds; Gold Bluff (**A**), Davison (**B**), Levee Soc (**C**), Stone Lagoon (**D**), Bald Hills (**E**), and Point Reyes (**F**). The grey shaded areas represent temporal variation in *r*_*max*_ and the strength of density dependence. This figure was created in RStudio (R Version 3.5.0; https://cran.r-project.org/bin/windows/base/old/3.5.0/).

The growth models estimated the mean *r*_*max*_ among herds to be 0.231 (95% credible interval = (0.151, 0.345)), and the standard deviation of *r*_*max*_ among herds to be 0.045 (0.002–0.223). The growth model with temporal variation in both *r* and *β* was selected for the five herds in RNSP (Table 1, Fig. 1). The growth model with temporal variation only in *β* was selected for the Point Reyes herd. Using the selected growth model for the ALE Reserve herd, we did not detect density dependence, so the estimate of *K* overlapped 0 (Table 2). *K* at or below 0 is not biologically realistic for a population that has existed for over 30 years^25^. These results may be a consequence of the ALE Reserve herd not yet reaching *K*. Nevertheless, because of the unrealistic parameter estimates, we omitted this herd from subsequent analyses.

**Table 1:** Mean deviance for each of the four possible Ricker growth models for each herd; no temporal variation in intrinsic population growth rate (*r*) or strength of density dependence (*β*), temporal variation in *r*, temporal variation in *β*, and temporal variation in both *r* and *β*.

Herd	No temporal variation	*r* temporal variation	*β* temporal variation	Both temporal variation
Gold Bluffs	− 131.83	− 158.99	− 150.08	− 167.70*
Davison	− 131.83	− 161.72	− 140.38	− 164.60*
Levee Soc	− 131.83	− 160.59	− 148.12	− 164.88*
Stone Lagoon	− 131.83	− 155.51	− 154.03	− 164.06*
Bald Hills	− 131.83	− 159.77	− 146.11	− 168.02*
Point Reyes	− 131.83	− 131.83	− 174.71*	− 169.92
ALE Reserve	− 131.83	− 155.26	− 144.48	− 165.74*

The asterisks denote the model with the lowest mean deviance by more than 2; this model was selected.

**Table 2:** Estimates (median) and 95% credible intervals of population growth parameters by the selected Ricker growth model for each herd.

Herd	*r*_*max*_	*r* temporal variation	*β*	*β* temporal variation	*K*
Gold Bluffs	0.243 (0.116, 0.439)	0.127 (0.008, 0.281)	− 0.01143 (− 0.02013, − 0.00431)	0.00489 (0.00028, 0.01192)	22 (14, 39)
Davison	0.226 (0.045, 0.365)	0.118 (0.006, 0.234)	− 0.00564 (− 0.00990, − 0.00064)	0.00170 (0.00008, 0.00542)	40 (24, 80)
Levee Soc	0.245 (0.102, 0.447)	0.084 (0.004, 0.173)	− 0.00494 (− 0.00909, − 0.00172)	0.00113 (0.00004, 0.00324)	50 (38, 75)
Stone Lagoon	0.256 (0.146, 0.543)	0.057 (0.003, 0.136)	− 0.00491 (− 0.00978, − 0.00268)	0.00097 (0.00005, 0.00237)	53 (42, 67)
Bald Hills	0.242 (0.147, 0.366)	0.072 (0.005, 0.162)	− 0.00091 (− 0.00151, − 0.00039)	0.00035 (0.00002, 0.00076)	267 (200, 444)
Point Reyes	0.226 (0.162, 0.320)	–	− 0.00060 (− 0.00102, − 0.00022)	0.00044 (0.00003, 0.00077)	380 (262, 832)
ALE Reserve	0.228 (0.136, 0.320)	0.090 (0.007, 0.203)	− 0.00032* (− 0.00089, 0.00026)	0.00042 (0.00005, 0.00114)	588* (− 4533, 5614)

The intrinsic population growth rate is *r* and strength of density dependence is *β.*

The asterisks denote 95% credible intervals that overlap 0.

**Figure 2:**
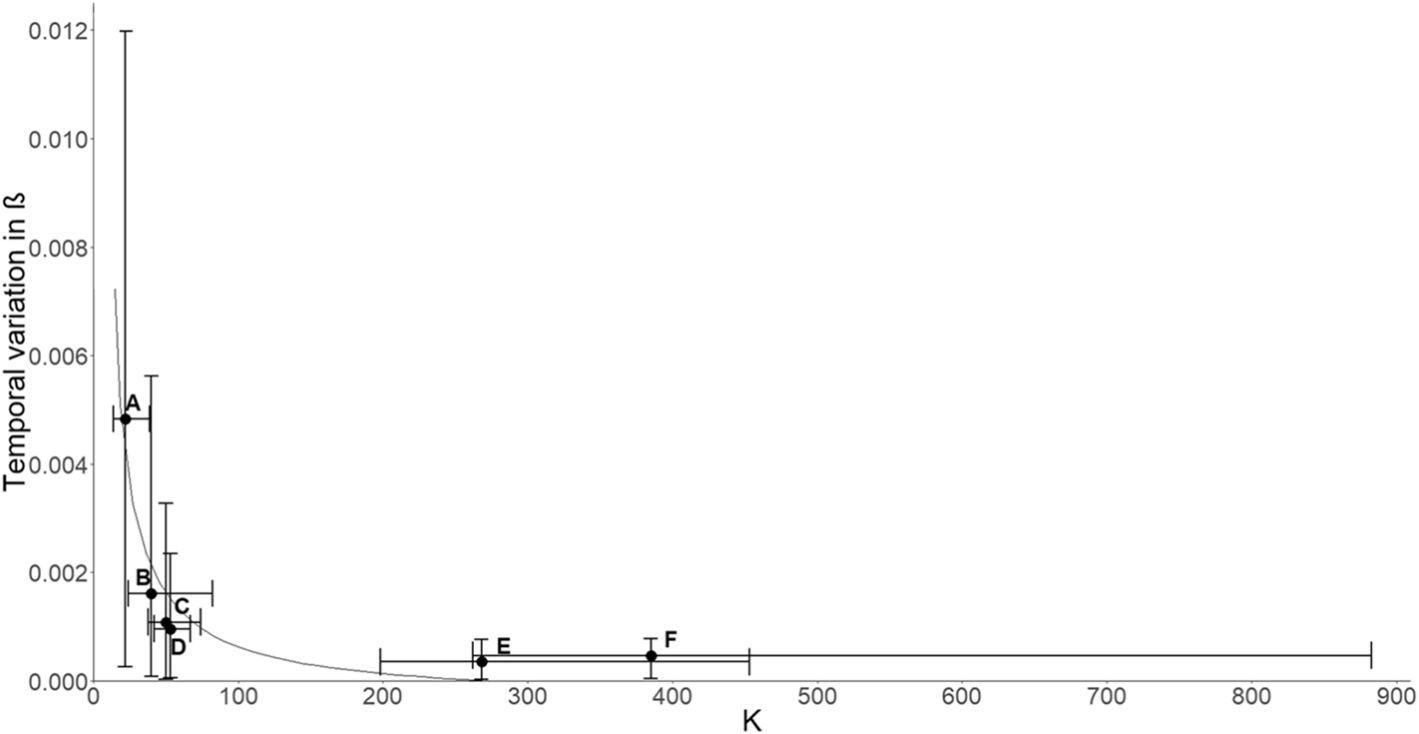
Temporal variation in strength of density dependence (*β*) and carrying capacity (*K*) for six elk herds. Letter designations are the same as in Fig. 4. A value for the density dependence further from zero indicates stronger density dependence. The estimated regression was $temporal\, variation\, in\, strength\, of \,density\, dependence = \frac{0.099}{K} - 0.00036$ (*R*^2^ = 0.87, P = 0.006). Error bars represent 95% credible intervals for *K* and temporal variation in the strength of density dependence. This figure was created in RStudio (R Version 3.5.0; https://cran.r-project.org/bin/windows/base/old/3.5.0/).

**Figure 3:**
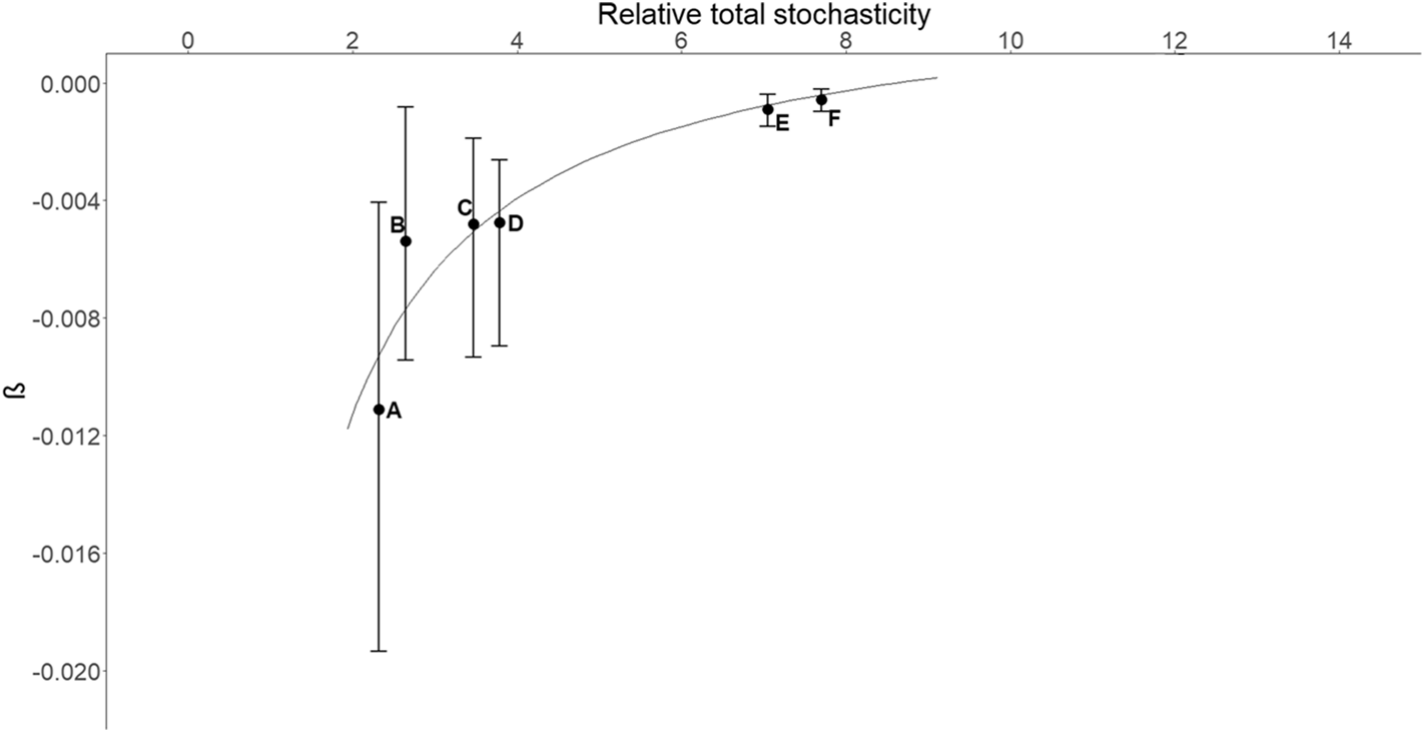
Strength of density dependence and relative total stochasticity for six elk herds. Letter designations are the same as in Fig. 4. A value for the strength of density dependence further from zero indicates stronger density dependence. The estimated regression was $density \,dependence = \frac{ - 0.0293}{{relative\, total \,variance}} + 0.0034$ (*R*^2^ = 0.88, P = 0.006). Error bars represent 95% credible intervals for density dependence. Our calculation of the relative total variance did not include an estimate of variance. This figure was created in RStudio (R Version 3.5.0; https://cran.r-project.org/bin/windows/base/old/3.5.0/).

Temporal variance in *β* was greater in herds with smaller *K* than larger *K*, and this relationship was also non-linear (Fig. 2). In other words, within herds, it appeared that a constant change in *K* affected temporal variation in *β* in herds with smaller *K* more than herds with larger *K*. A positive, non-linear correlation was detected between *β* and the relative total stochasticity (Fig. 3), such that herds with smaller *K* experienced less fluctuation in abundances than herds with larger *K*. For the relative influence of demographic, density-dependent, and density-independent sources of stochasticity, see Supplementary Information Fig. S2.

## Discussion

Variation in *K* and the consequences to population dynamics have usually been examined among populations^18^ or within populations^21^. We simultaneously examined variation in *K* both within and among populations. At the same time, we also examined temporal variation in *r* within populations. Our findings indicated that population growth parameters varied within and among populations. As expected, we found that *K* and *β* varied among populations and that there was less fluctuation in abundance around *K* when *K* was lower. Surprisingly, *β* varied temporally in all six populations, and *r* varied temporally in five populations. In other words, whereas variation in most populations have been explained by either the additive or the multiplicative model^9^, we found that both types of variation were displayed in five out of six elk populations.

Variation in *K* and *β* is an important consideration in population regulation, especially in the face of current environmental change^5,8^. As we had expected, we found variation in *K* among herds within a small part of the species’ geographic range, but we also unexpectedly found variation in *K* within herds. This result indicates that assuming constant *K* across time is probably unrealistic, even when environments seem to be mild and stable. As such, in order to fully understand population dynamics and regulation, parameters that can accommodate stochastic environments should be incorporated into models^5,21^. Considering temporal variation in *K* in population projection is necessary to encompass all possibilities for the population’s growth, although it may result in more uncertainty around predictions.

Environmental heterogeneity and variation in *K* among populations is expected across a species’ geographic range^18,22^. Our findings, which were from herds with clear spatial boundaries, suggest that similar variation in *K* among herds can manifest within a small part of the species’ geographic range. Within RNSP, forage supply varies both across herd home ranges and temporally, which can affect *K*^7^. Also, social dynamics between herds can impact elk movement and *K*. For example, meadow partitioning in RNSP is presumably enforced by social fences between herds (i.e., when elk avoid interacting with unfamiliar individuals from other herds). This meadow partitioning can dictate the amount of forage available to each herd and, consequently, *K* and *β*^7^. These findings indicate that variation in *K* is not solely coupled to environmental variables that reflect food supply and other habitat attributes critical to survival and reproduction. Rather, a social dimension can generate variation in *K*, both among populations and temporally within populations. This result highlights the importance of considering social aspects, not just variation in resource availability, for management decisions, such as identifying optimal harvest. Furthermore, as *K* varies temporally within populations, optimal harvest will likely also vary across time.

Stronger density dependence in herds with smaller *K* resulted in less stochastic fluctuations in abundance than herds with larger *K*. Strong direct (i.e., not lagged) density dependence was also shown to increase stability in several elk populations in the northern hemisphere^8^. However, herds with smaller *K* had higher temporal variation in *β*. In other words, for the same change in *K* over time, *β* will change more in a herd with smaller *K* than one with larger *K*. Temporal variation in *β* can be generated by fluctuations in the amount or quality of resources, which in turn drives fluctuations in the strength of competition among individuals for resources^9^. It can lead to growth-catastrophe dynamics, where periods of weak density dependence followed by periods of strong density dependence can cause abundances to rapidly decline^7,9^.

Given that variation in *β* is often described as important for understanding return time to equilibrium^5,8^, these findings have implications for population and land management. For example, increasing available resources, such as forage, for a herd will increase its *K* and decrease *β*, which, as our results indicate, may cause the population to experience less stability. Changes in resource availability can occur by several means. For example, temporal variation in *K* likely occurred in the Davison herd when the land area available to the herd for foraging increased when cattle were removed from a meadow in 2016^7,26^. In contrast, the area available for foraging to the Point Reyes herd could not change because of a fence that restricted the herd to a limited area. Nonetheless, annual precipitation in Point Reyes was highly variable and was correlated with calf recruitment^27^. Thus, variation in precipitation likely resulted in variation in the amount of forage available and, therefore, temporal variation in *K*. Increasing available resources or introducing a population to a new range may even lead to an irruption and overshoot of a herd’s food supply^7,28–30^. Our results suggest that populations with smaller *K* are more vulnerable to destabilizing dynamics. This is because a constant change in *K* should affect *β* and temporal variation in *β* more dramatically in herds with smaller *K*.

Our estimate of *r*_*max*_ was similar to estimates of *r*_*max*_ for other elk populations^31–33^. Surprisingly, we found that *r* varied temporally within some herds. Temporal variation in *r* was detected in RNSP herds, but not in the Point Reyes herd, and the amount of temporal variation in *r* did not appear to be related to *K*. The within-herd temporal variation in *r* in RNSP herds was unexpected considering that this study area had a stable habitat composition and mild climate. Furthermore, density-dependent climatic factors did not influence juvenile recruitment in the Davison herd^7,30^. While density-independent factors are often described as climatic factors^34^, they can in fact be a number of abiotic factors^8^, and can even include movement of individuals into or out of a population. Weckerly^7^ describes temporal variation in *r* due to immigration in the Davison herd. In contrast, temporal variation in *r* in the Point Reyes herd was not detected. This may be because this herd also experienced a relatively stable and mild climate, and the elk were restricted on the peninsula by a well-maintained fence. Consequently, individual immigration and emigration was unlikely in this herd^27,35^. Therefore, neither climatic factors nor elk movement generated temporal variation in *r* in this herd. Populations which are restricted by a fence or are otherwise isolated from other conspecific populations should be expected to have less temporal variation in *r* because immigration or emigration will likely not occur. Furthermore, estimating *r* may be a method to determine whether net immigration into a population is occurring; if a population’s *r* is higher than *r*_*max*_ for the species, immigration is likely occurring.

Our findings add dimensions to understanding population regulation. Some of our findings reinforce what has been previously shown; variation in *K* among populations affected *β* and fluctuation in abundances around *K*. The added dimension of temporal variation in *K* within populations revealed that the amount of variability in *β* within a population is also affected by median or average *K*. Populations with small *K* can have greater variation in *β*, which has consequences for population stability. Furthermore, population regulation is often understood in terms of the rate of return to the equilibrium abundance^1^, which is calculated using *β*. However, its interaction with temporal variation in *r* might complicate the relationship between *K*, *β*, and regulation. Therefore, considering temporal variation in population growth parameters is also necessary to understand population regulation.

## Methods

### Study areas

**Figure 4:**
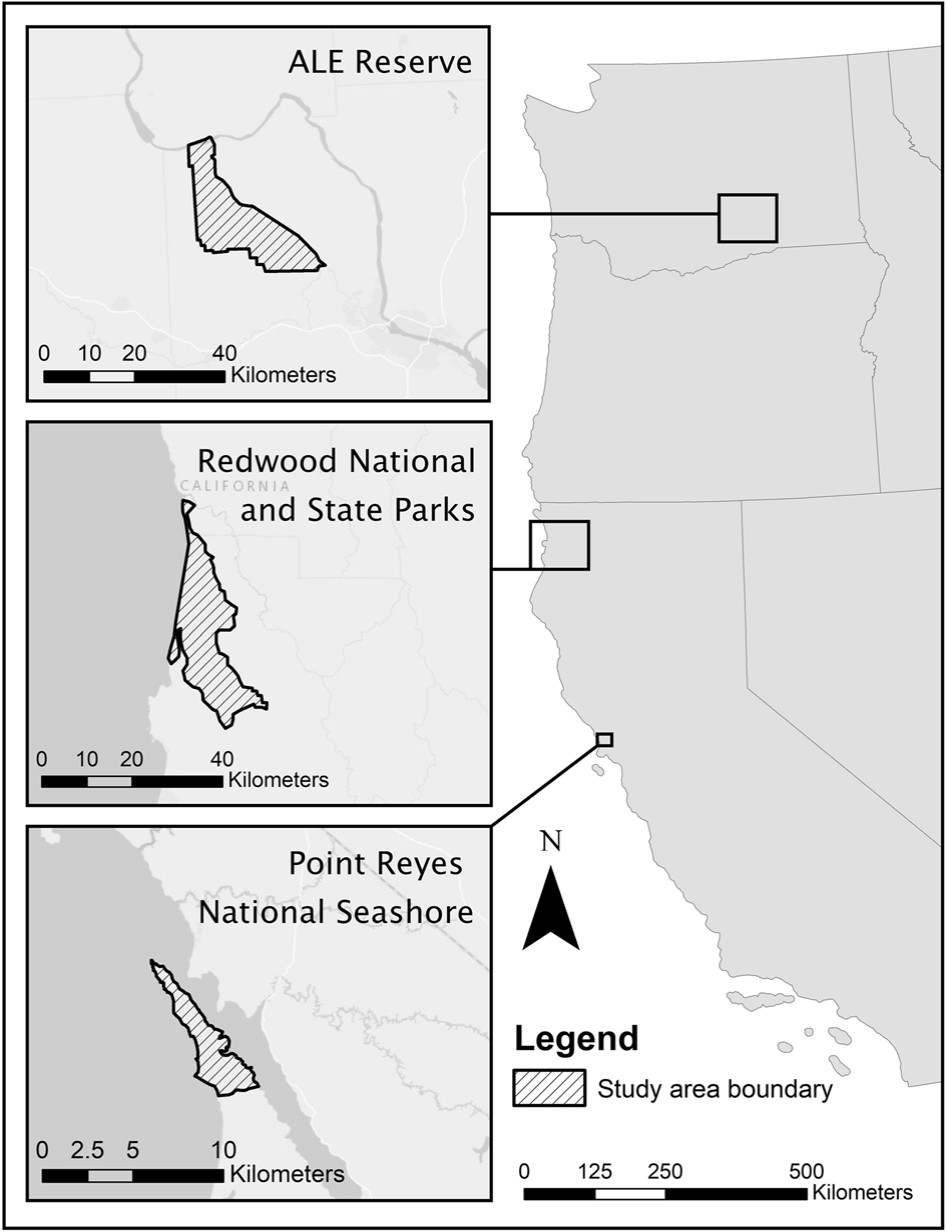
Map of study areas in Arid Lands Ecology (ALE) Reserve, southern part of Redwood National and State Parks, and Tomales Point Elk Reserve in Point Reyes National Seashore. This map was created in ArcMap (Version 10.6; https://desktop.arcgis.com/en/arcmap/).

Time series of population survey data were used from nonmigratory elk populations in three different locations along the West Coast of the USA (Fig. 4). Five of the populations were in the Prairie Creek drainage (Davison), the Lower Redwood Creek drainage (Levee Soc), the Stone Lagoon area, the Gold Bluffs region, and the Bald Hills region of Redwood National and State Parks (RNSP), Humboldt County, California (41.2132° N, 124.0046° W). These populations occupy an area of about 380 km^2^. The climate in this region was mild, with cool summers and rainy winters. Annual precipitation was usually between 120 and 180 cm and most of the precipitation fell between October and April. Snow was rare since average winter temperatures rarely dropped below freezing and ranged from 3 to 5 °C. Average summer temperatures ranged from 10 to 27 °C, depending on the distance inland. Elk in RNSP were not legally hunted, and displayed strong social bonding between females, juveniles, and sub-adult males^7^.

An elk population in the Point Reyes National Seashore inhabited part of the Point Reyes Peninsula in Marin County, California (38.0723° N, 122.8817° W). The elk were restricted to an area of 10.52 km^2^ on the northern tip of the peninsula by a 3-m-tall fence. The climate of this study area was Mediterranean, with an average annual precipitation of 87 cm^27^. Most of the precipitation fell from autumn to early spring. Temperatures averaged about 7 °C in winter and 13 °C in summer^27,35^.

Another elk population was in the Arid Lands Ecology (ALE) Reserve and occupied a 300 km^2^ area within the U.S. Department of Energy’s Hanford Site, Washington (46.68778° N, 119.6292° W). The climate in this area was semi-arid with dry, hot summers and wet, moderately-cold winters. Average summer temperatures were around 20 °C and average winter temperatures were around 5 °C with an average annual precipitation of 16 cm, half of which fell in the winter as rain^36^.

### Population surveys

In RNSP, females, juveniles, and subadult males were often in the same group and tended to use open meadow habitat more frequently than adult males^37,38^. These behavioral patterns likely explain why females, juveniles, and subadult males were sighted more frequently than adult males^7^. Moreover, in size-dimorphic ungulates such as elk, recruitment was strongly correlated with female abundance and weakly correlated with male abundance^7,13,39^. In RNSP, the abundance of groups of females, juveniles, and subadult males drove the dynamics of the group and of adult males^7^. Therefore, for the RNSP populations, we used herd counts where a herd was comprised of females, juveniles, and subadult males. We also used herd counts for the Point Reyes and ALE Reserve populations to remain consistent.

Systematic herd surveys of elk were conducted during January from 1997 to 2019 in RNSP. Surveys in the Davison meadows, the Levee Soc area, the Stone Lagoon area, the Gold Bluffs region, and the Bald Hills region were conducted by driving specified routes 4 to 10 times on different days throughout the month of January. The time series for these five herds ranged from 19 to 23 years of data. The elk were counted and classified by age and sex as adult males, subadult males, females, and juveniles. Females could not be visually differentiated into adult and subadult age categories^37^. The highest count of females, juveniles, and subadult males from the surveys conducted each year was used as an index of abundance of each herd since the detection probabilities were high both on an absolute basis (> 0.8) and relative to variation in detection probabilities (CV_sighting_ = 0.05)^7,40^. For the Bald Hills herd, which is the only herd in RNSP where harvests occurred, we added hunter harvests to the highest count of each year to account for this source of mortality. These harvests occurred only when elk from the Bald Hills herd left RNSP.

Elk population surveys were conducted at the Point Reyes National Seashore from 1982 to 2008. Weekly surveys were conducted after the mating season. Surveys were conducted on foot or horseback of female elk that were ear-tagged or had a collar containing radio telemetry^32,35^. Individuals counted were classified as females, juveniles, subadult males, and adult males. Data were not available for the years 1984 to 1989 and 1993, so the time series included 20 years of data. We used the highest count of females, juveniles, and subadult males in each year in our analyses. This herd was also not hunted.

Elk population surveys in the ALE Reserve were conducted in winters after hunting and before parturition. From 1982 to 2000, biologists used aerial telemetry studies, in which they located all collared elk during each survey and classified them by sex and age. We used the total counts of females, juveniles and subadult males. For years in which multiple surveys were conducted, we used the highest count in each year as an index of abundance for that year^25,41^. We omitted population survey data collected in 1982 from our analysis because individuals were not classified by sex and age in this year. Consequently, the time series included 18 years of data. For all years of data used, we added hunter harvests to the highest count of each year to account for this source of mortality. The count in 2000 was much lower than in the previous year, likely due in part to a large wildfire which occurred in the summer of 2000, which probably had an immediate effect of reducing available elk forage in the reserve and caused elk to spend more time outside of the ALE Reserve^42,43^. In addition, the highest recorded number of elk (about 291) were harvested that year^43^.

### Ricker growth models

We fit linearized Ricker growth models simultaneously to the seven time series to estimate population growth parameters as well as temporal variation in *r* and *β*. We estimated *K* as the x-intercept of the Ricker growth model (i.e., when *r* = 0). Notably, preliminary analyses showed that not accounting for observer error did not bias our results (see Supplementary Information).

We used a Bayesian Markov Chain Monte Carlo (MCMC) algorithm with 3 chains, 150,000 iterations, a burn-in period of 75,000, an adaptation period of 75,000, and no thinning. We used Bayesian inference and MCMC because these methods offer advantages when fitting hierarchical models to model variation in ecological data^44,45^. We conducted these analyses in the RJAGS program (JAGS Version 4.0.0; https://sourceforge.net/projects/mcmc-jags/files/JAGS/4.x/Windows/) in RStudio (R Version 3.5.0; https://cran.r-project.org/bin/windows/base/old/3.5.0/). We used uninformative priors for the y-intercept (i.e., *r*_*max*_) and the slope (i.e., *β*) in order to allow solely the data to influence posterior estimates of these parameters. Informative priors were not necessary as long as parameter estimates from each chain converged. Convergence among chains was determined when the Gelman-Rubin diagnostic ($\hat{R}$) was less than 1.01, and through visual checks of trace and density plots^46^.

The estimate of *r*_*max*_ borrowed information among herds because this parameter should be similar among populations within a species^22^. Therefore, we modeled *r*_*max*_ for each herd (*j*) as a random effect following a normal distribution with $\mu_{{r_{max} }} \sim Normal\left( {0, 0.001} \right)$ and $\sigma_{{r_{max} }} \sim Uniform\left( {0, 100} \right)$. To model temporal variation in *r* for each herd, we included a zero-centered random effect which was also modeled following the normal distribution $\gamma_{t,j} \sim Normal\left( {0,\sigma_{{\gamma_{j} }} } \right)$, where $\sigma_{{\gamma_{j} }} \sim Uniform\left( {0, 100} \right)$. The estimate of *β* did not borrow information among herds because this parameter can vary widely among herds^18^. The prior for *β* for each herd (*j*) followed the normal distribution $\beta_{j} \sim Normal\left( {0, 0.001} \right)$. To model temporal variation in *β* for each herd, we modified how we modeled *β* by using a normal distribution ${\beta_{{\delta }_{t,j}}} \sim Normal\left( {\mu_{{\beta_{{\delta }_{j} }}}} , {\sigma_{{\beta_{{\delta }_{j}} }} } \right)$, where ${\mu_{{\beta_{{\delta }_{j}} }}} \sim Normal\left( {0, 0.001} \right)$ and ${\sigma_{{\beta_{{\delta }_{j}} }}} \sim Uniform\left( {0, 100} \right)$. Thus, there were four possible Ricker growth models for each herd; (1) no temporal variation in *r* and *β*,

3 $$ r_{t} = r_{max} + \beta N_{t} + \varepsilon , $$

(2) temporal variation in *r*,

4 $$ r_{t} = r_{max} + \beta N_{t,} + \gamma_{t} + \varepsilon , $$

(3) temporal variation in *β*,

5 $$ r_{t} = r_{max} + {\beta_{{\delta }_{t}}} N_{t} + \varepsilon , $$

and

(4) temporal variation in both *r*_*max*_ and *β*

6 $$ r_{t} = r_{max} + {\beta_{{\delta }_{t}}} N_{t} + \gamma_{t} + \varepsilon . $$

The residual variance was modeled as $\varepsilon \sim Uniform\left( {0,100} \right)$. We fit the model with no temporal variation (Eq. (3)) in either parameter to all seven time series simultaneously. All parameters except for *r*_*max*_ were estimated independently for each herd. For each time series of population survey data, we determined whether models with more parameters provided a better fit. We did so by fitting each possible growth model (Eqs. (4)–(6)) to each time series one at a time, while modeling all other time series with no temporal variation in *r*_*max*_ or *β* (Eq. (3)). The model with the lowest mean deviance from RJAGS by more than 2 was selected for that herd^47^.

### Environmental and demographic stochasticity

We estimated fluctuation in abundance which can be attributed to demographic and environmental stochasticity for herds with different *K* for each herd. The stochasticity model was outlined by Ferguson and Ponciano^9^;

7 $$ Var\left( {N_{t - 1} } \right) = Var_{dem} \left( {N_{t - 1} } \right) + Var_{r} \left( {N_{t - 1} } \right) + Var_{{\upbeta }} \left( {N_{t - 1} } \right), $$

where $Var\left( {N_{t - 1} } \right)$ was total population stochasticity, $Var_{dem} \left( {N_{t - 1,} } \right)$ was population abundance fluctuation due to demographic stochasticity, $Var_{r} \left( {N_{t - 1} } \right)$ was population abundance fluctuation due to changes in *r* (i.e., density-independent environmental stochasticity), and $Var_{\beta } \left( {N_{t - 1} } \right)$ was population abundance fluctuation due to changes in *β*. The model assumes density-dependent survival following the Ricker model. Demographic stochasticity was calculated as follows;

8 $$ Var_{dem} \left( {N_{t - 1} } \right) = \alpha N_{t - 1} e^{{ - \beta_{\Delta } \left( {N_{t - 1} } \right)}} \left( {1 - e^{{ - \beta_{\Delta } \left( {N_{t - 1} } \right)}} } \right) + \sigma_{dem}^{2} N_{t - 1} e^{{ - 2\beta_{\Delta } \left( {N_{t - 1} } \right)}} $$

where $\sigma_{dem}^{2}$ was assumed to be equal to *α*^9^. Environmental stochasticity that is expressed as changes in *r*, otherwise known as density-independent or additive stochasticity, was calculated as follows;

9 $$ Var_{r} \left( {N_{t - 1} } \right) = \sigma_{{\beta_{\Delta } }}^{2} \alpha^{2} N_{t - 1}^{2} e^{{ - 2\beta_{\Delta } \left( {N_{t - 1} } \right)}} , $$

and environmental stochasticity that is expressed as changes in *β*, otherwise known as density-dependent or multiplicative stochasticity, was calculated as follows;

10 $$ Var_{{\upbeta }} \left( {N_{t - 1} } \right) = \sigma_{{\beta_{\Delta } }}^{2} \alpha^{2} N_{t - 1}^{2} \left( {N_{t - 1} } \right)^{2} e^{{ - 2\beta_{\Delta } \left( {N_{t - 1} } \right)}} . $$

Population growth parameters from the selected Ricker growth model for each herd were used in these equations to estimate each of these sources of stochasticity for each herd across abundances ranging from five to above *K*. The relative total population stochasticity was expressed as the total population stochasticity at *K* for each herd divided by that herd’s *K*.

## Supplementary information

Supplementary Information.

## Data Availability

The datasets analyzed in our study will be made available in an online data repository.
