# Supplementary material for: Elk population dynamics when carrying capacities vary within and among herds
Source: Sci Rep. 2020 Sep 29;10:15956. doi: 10.1038/s41598-020-72843-5 (PMC7524762; doi:10.1038/s41598-020-72843-5)
Supplement: Supplementary file 1 — Supplementary Information. [file 41598_2020_72843_MOESM1_ESM.docx]

Elk Population Dynamics When Carrying Capacities Vary Within and Among Herds

Lisa J. Koetke, Adam Duarte, Floyd W. Weckerly

**SUPPLEMENTARY INFORMATION**

**METHODS**

*State-Space Models*

Since the Ricker growth models described in this paper do not account for observer error, we used hierarchical models in a state-space formulation to distinguish observer error (i.e., measurement and sampling errors) from process variance in herd counts ^1-3^. We then compared the results of the growth models with the state-space model to verify that observer error was not biasing my results. We modeled observer error using a Poisson distribution such that $N_{o_{ij}}\sim Poisson(N_{e_{ij}})$, where $N_{o_{ij}}$ is the count in year *i* from the survey of herd *j* and $N_{e_{ij}}$ is the estimated true abundance from year *i* of herd *j*. We selected the Poisson distribution since larger herds tend to increase the probability of double-counting individuals or missing individuals that were obscured by other animals, vegetation, or terrain ^4^.

I fit the Ricker model using Bayesian statistics and Markov Chain Monte Carlo algorithms with three chains, 150,000 iterations, a burn-in period of 75,000, an adaptation period of 75,000, and no thinning. Convergence among chains was determined by whether $\hat{R}<1.01$ and visual checks of trace and density plots ^5^. We used uninformative priors for *r_max_* and *K*. We modeled *r_max_* for each herd as a random effect following the normal distribution $r_{max}\sim Normal(\mu_{r_{max}},\sigma_{r_{max}})$, where $\mu_{r_{max}}\sim Normal(0, 0.001)$ and $\sigma_{r_{max}}\sim Uniform(0, 100)$. We modeled *K* using a gamma distribution (shape = 0.01, rate = 0.01). When estimating the models, we drew the population abundance in the first year from a Poisson distribution with stochasticity included, such that $N_{e_{1j}}\sim Poisson(\lambda)$ and $\lambda=Uniform(N_{o_{1j}}-5,N_{o_{1j}}+5)$ where $N_{e_{1j}}$ was the estimated abundance in the first year of herd *j* and $N_{o_{1j}}$ was the count for the first year of herd *j*. If $N_{o_{1j}}-5<1$, it was rounded to 1. We used this initial abundance of each herd to estimate abundance in year *i* ${(N}_{e_{ij}})$ for the next $n_{j}$ years, where $n_{j}$ is the number of years of population survey data for herd *j*, using the Ricker model.

**RESULTS**

*State-Space Models*
The state-space model estimated the mean *r_max_* among herds to be 0.231 (95% credible interval = [0.091, 0.348]), and the standard deviation of *r_max_* among herds to be 0.081 (95% CI = [0.006, 0.295]). For parameter estimates, see Table A1.

The 95% credible intervals of parameters estimated by the Ricker growth models (Table 2) and the Ricker state-space model (Appendix, Table A1) overlapped. This finding indicates that the results of the growth models were similar to the results of fitting the state-space models, which distinguished observer error from process variance. Furthermore, the 95% credible intervals of each of the abundance estimates of the Ricker state-space model overlapped the abundance survey data, except one year for the Bald Hills herd, three years in the Point Reyes herd, and two years in the ALE Reserve herd (Appendix, Fig. A1). Thus, because the state-space model was in agreement with the data and the growth models were in agreement with the state-space model, the growth model estimates were likely to be little biased by observer error.

**Table S1.** Estimates (median) and 95% credible intervals of the maximum intrinsic growth rate (*r_max_*) and carrying capacity (*K*) by the state-space model for each herd.

| Herd | *r_max_* | *K* |
| --- | --- | --- |
| Gold Bluffs | 0.233  [0.011, 0.444] | 23  [11, 40] |
| Davison | 0.185  [-0.030, 0.328] | 44  [13, 126] |
| Levee Soc | 0.223  [-0.011, 0.447] | 51  [21, 98] |
| Stone Lagoon | 0.249  [0.024, 0.540] | 53  [31, 70] |
| Bald Hills | 0.202  [0.120, 0.277] | 266  [229, 338] |
| Point Reyes | 0.248  [0.176, 0.323] | 366  [318, 446] |
| ALE Reserve | 0.274  [0.214, 0.341] | 630  [530, 794] |


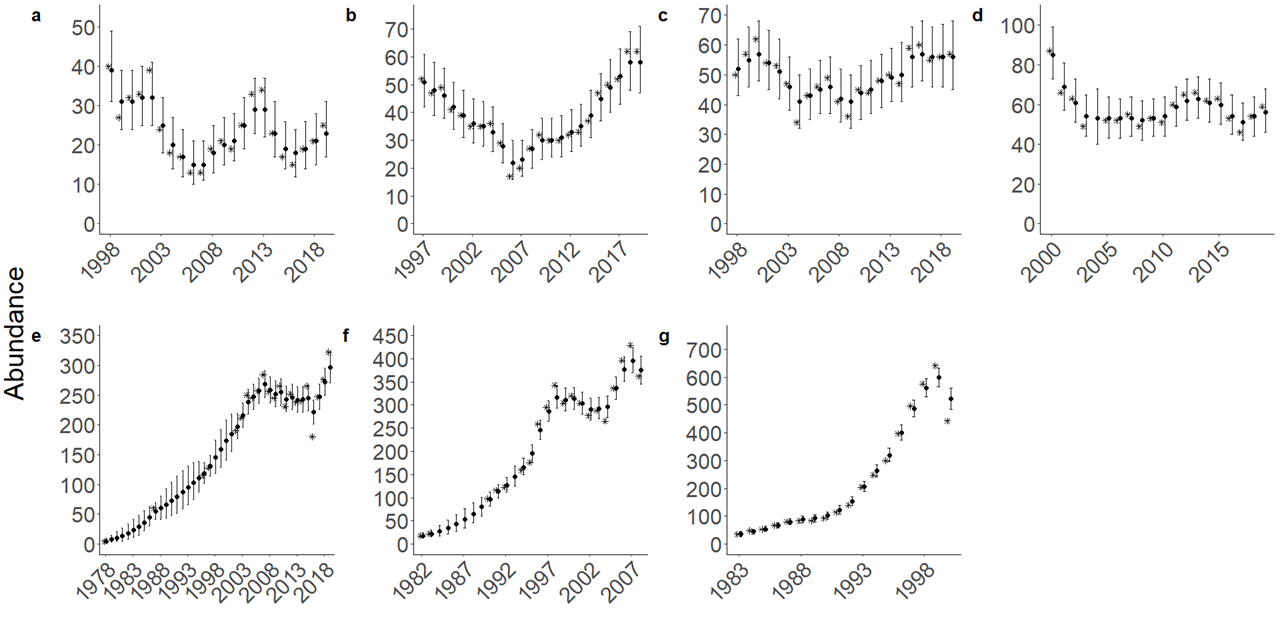


**Figure S1.** Population survey data (stars) and estimates of abundance from the Ricker model (circles) for seven elk herds; Gold Bluff (A), Davison (B), Levee Soc (C), Stone Lagoon (D), Bald Hills (E), Point Reyes (F), and ALE Reserve (G). Error bars represent the 95% credible intervals for each abundance estimate. This figure was created in RStudio (R Version 3.5.0; https://cran.r-project.org/bin/windows/base/old/3.5.0/).


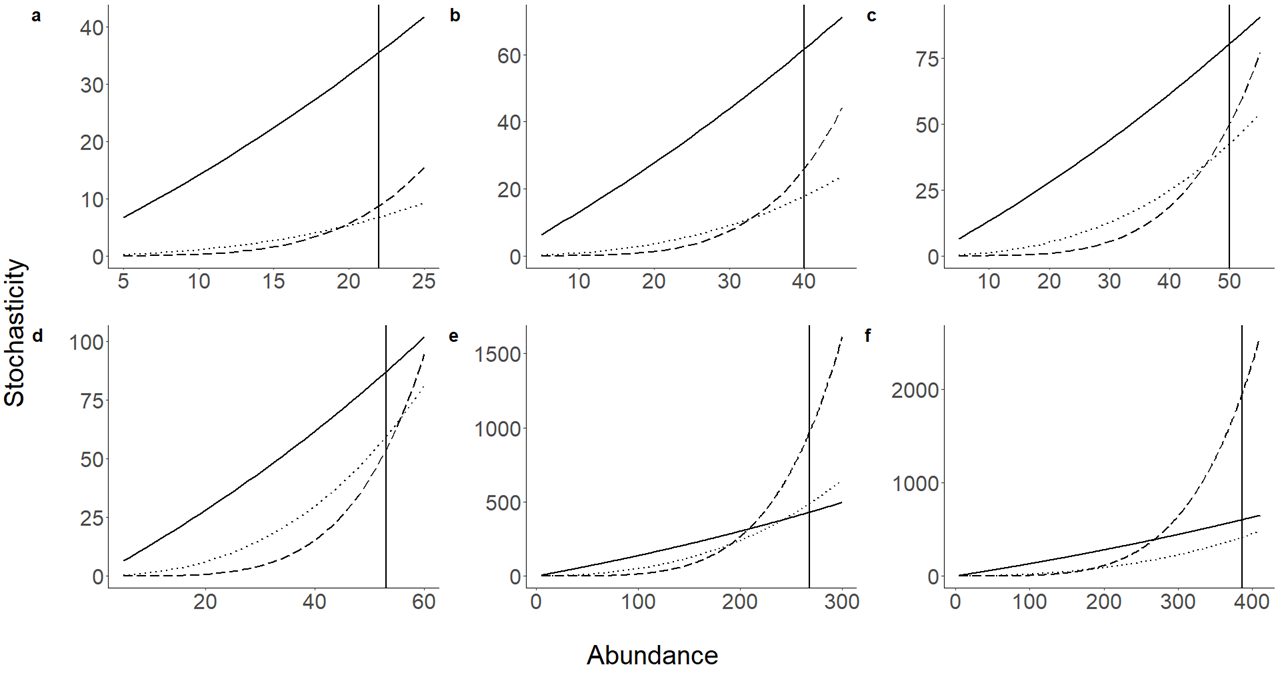


**Figure S2.** Predicted demographic variation (solid), variation in strength of density dependence (*β*; dashed), and variation in population intrinsic growth rate (*r*; dotted) for six elk herds. Letter designations are the same as in Figure S1. The vertical line represents the estimated carrying capacity (*K*) of each herd. This figure was created in RStudio (R Version 3.5.0; https://cran.r-project.org/bin/windows/base/old/3.5.0/).

**REFERENCES**

1 Clark, J. S. & Bjørnstad, O. N. Population time series: Process variability, observation errors, missing values, lags, and hidden states. *Ecology* **85**, 3140–3150 (2004).

2 de Valpine, P. & Hastings, A. Fitting population models incorporating process noise and observation error. *Ecological Monographs* **72**, 57–76 (2002).

3 de Valpine, P. Better inferences from population-dynamics experiments using monte carlo state-space likelihood methods. *Ecology* **84**, 3064–3077 (2003).

4 Weckerly, B. *Population ecology of Roosevelt elk: conservation and management in Redwood National and State Parks*. (University of Nevada Press, 2017).

5 Kery, M. *Introduction to WinBUGS for ecologists*. (Academic Press, 2010).
